# A Starch Phosphorylase, ZmPHOH, Improves Photosynthetic Recovery from Short-Term Cold Exposure in Maize

**DOI:** 10.3390/ijms26041727

**Published:** 2025-02-18

**Authors:** Yao Qin, Haiping Ding, Hailiang Zhao, Xueqing Zheng, Jing Wang, Ziyi Xiao, Yuanru Wang, Hongwei Wang, Yinggao Liu, Dianming Gong, Fazhan Qiu

**Affiliations:** 1 Hubei Hongshan Laboratory, National Key Laboratory of Crop Genetic Improvement, Huazhong Agricultural University, Wuhan 430070, China; 15207134420@163.com (Y.Q.);; 2 State Key Laboratory of Wheat Breeding, College of Agronomy, Shandong Agricultural University, Taian 271018, China; 3 State Key Laboratory of Crop Biology, College of Life Sciences, Shandong Agricultural University, Taian 271018, China

**Keywords:** maize, starch phosphorylase 2, cold stress, metabolome profiling, photosynthetic recovery

## Abstract

The photosynthetic system of maize (*Zea mays*) leaves is sensitive to low temperatures and suffers from irreversible damage induced by cold exposure, making cold stress a major factor limiting maize yield. Identifying genes that improve the recovery of photosynthesis from low temperatures in maize will help enhance the cold tolerance of this crop and ensure stable yields. Here, we demonstrate the role of *starch phosphorylase 2* (*ZmPHOH*) in promoting photosynthetic recovery from cold damage. *Chlorotic leaf3* (*chl3*), a null mutant of *ZmPHOH*, which undergoes chlorophyll degradation and chlorosis earlier than under normal growth conditions after brief exposure to 8 °C and restoration to normal. We determined that *chl3* plants could not repair the damage to their photosynthetic system caused by short-term cold exposure after the temperature returned to normal. Metabolome and transcriptome profiling indicated that the soluble sugar content in *chl3* leaves was significantly increased after cold treatment and could not be catabolized promptly, leading to repression of photosynthetic gene expression. Our results reveal that ZmPHOH enhances post-cold photosynthetic recovery by promoting the decomposition and metabolism of soluble sugars, thereby regulating the low-temperature resilience in maize, which provides new insights into the chilling tolerance mechanism of maize.

## 1. Introduction

Cold stress affects crop growth and productivity worldwide. Maize (*Zea mays*) is widely used worldwide in the production of food, feed, and biofuels. The tropical origin of maize underlies its greater sensitivity to low temperatures compared to other crop species; however, more than 60% of its production area is located in countries with temperate climates [1]. In these areas, maize seedlings frequently experience chilling damage in early spring. As a C4 plant, maize achieves a higher photosynthetic efficiency than C3 crop species but performs poorly when exposed to low temperatures. Indeed, its photosynthetic system is susceptible to low temperatures (5–15 °C), and even when conditions return to normal, the damaged photosynthetic capacity is difficult to recover [2,3]. Exposure to low temperature not only causes damage to chloroplast structures but also inhibits the transcription of genes encoding key enzymes in photosynthesis, such as *RbcL* and *RbcS*, genes encoding photosystem proteins, such as *PsbA* and *PsbD*, and genes related to chlorophyll synthesis, such as *CHLH* and *PORA*, ultimately affecting the growth and development of maize and leading to a yield reduction of 5–30% [4,5,6]. Thus, a thorough understanding of cold stress–tolerance mechanisms in maize is needed to overcome the limitations imposed by cold and improve maize yield, especially in terms of restoring the photosynthetic capacity of maize plants after cold exposure.

Low temperature induces a burst of reactive oxygen species (ROS), leading to membrane lipids peroxidation, changes in protein activity, and decreased photosynthetic capacity [7]. To survive under such unfavorable conditions, plants need to maintain cellular functions and integrity by stabilizing cell membranes and biologically active proteins to sustain basic physiological activities [8]; optimizing carbohydrate metabolism is an effective means to resist cold stress. Under these challenging environmental conditions, plants generally remobilize transitory starch to provide an energy source and carbon backbones when their production via photosynthesis may be limited [9,10]. In Arabidopsis (*Arabidopsis thaliana*), mutation of *α-GLUCAN WATER DIKINASE 1* (*GWD1*, also reported as *STARCH EXCESS 1* [*SEX1*]), encoding an enzyme catalyzing the first step of starch degradation, results in decreased chilling tolerance. The cold-sensitive phenotype can be rescued by overexpressing the cDNA of *GWD1* in the mutant background, suggesting that the *GWD1* locus is associated with cold tolerance in Arabidopsis [11]. *β-AMYLASE 8* (*BAM8*), the enzyme acting downstream of GWD1, accelerates the degradation of starch to maltose and regulates cold tolerance in Arabidopsis, with *BAM8* expression being significantly induced by exposure to low temperatures [12]. When the Arabidopsis starchless mutant *plastidial phosphoglucose mutase1* (*pgm1*) is subjected to low temperature (5 °C), its leaves are unable to accumulate maltose and raffinose family oligosaccharides (RFOs), and it accumulates much lower levels of hexoses than the wild type, ultimately leading to diminished chilling tolerance [13]. A function for BAMs in response to cold stress has also been demonstrated in rice (*Oryza sativa*), trifoliate orange (*Poncirus trifoliata*) and *Medicago ruthenica* by promoting starch degradation [14,15,16]. These results have suggested that the degradation of transitory starch is an important source of osmoprotective substances (soluble sugars) and that blocking this reaction may lead to a decreased plant tolerance to low temperatures. In addition, promoting starch degradation is an important part of the plant response to cold stress, and different plants may also use this strategy.

Higher soluble sugar levels contribute to plant survival during their cold response; however, they are not beneficial to plant growth under normal conditions. Indeed, excessive levels of soluble sugars inhibit the transcription of photosynthetic genes and decrease the efficiency of photosynthesis, also affecting normal plant growth and development [17,18]. Previous studies have shown that many mutants with high soluble sugar levels due to metabolic or transport defects exhibit phenotypes such as chlorotic leaves, inhibition of plant growth, and lower fertility [19,20,21]. Although carbohydrate metabolism during the cold response is generally better understood, how altered carbohydrate levels caused by exposure to chilling return to normal levels after resumption of normal temperature conditions is still unclear.

Few studies have focused on the genes related to soluble sugar metabolism in plants after return to normal growing conditions following cold exposure. For example, galactosidases were shown to play roles in the catabolism of RFOs in cucumber (*Cucumis sativus*) during the post-cold recovery [22], but the catabolism of other sugars and whether and how they participate in the post-cold response are still poorly understood. Thus, dissecting the mechanisms and related genes for their manipulation should facilitate alleviating the inhibition of photosynthesis caused by high levels of soluble sugars, promote the recovery of photosynthetic capacity after cold stress, and improve plant tolerance to low temperatures.

Here, we report a function for starch phosphorylase 2 (ZmPHOH) from maize in removing soluble sugars to promote the restoration of photosynthetic capacity during post-cold recovery. ZmPHOH was previously reported to be involved in maltose degradation, based on the characterization of its null mutant *chlorotic leaf3* (*chl3*), which exhibits chlorotic leaves and impaired photosynthesis after the five-leaf stage caused by excessive accumulation of carbohydrates in its leaves [23]. Returning *chl3* seedlings to normal growth temperature after 2 days of cold treatment led to leaf chlorosis before the five-leaf stage, together with accumulation of soluble sugars, causing suppression of photosynthetic gene expression and sustained inhibition of photosynthesis, thus adversely affecting later growth and development. Our study elucidates how carbohydrate metabolism influences the recovery of photosynthesis in maize following chilling exposure, and revaled the irreplaceable role of ZmPHOH in this process. These results not only deepen our understanding of carbohydrate metabolism during post-cold recovery but also provide a theoretical basis for genetic improvement of maize chilling tolerance.

## 2. Results

### 2.1. Low Temperature Induces a Premature Chlorosis Phenotype in chl3

**Figure 1:**
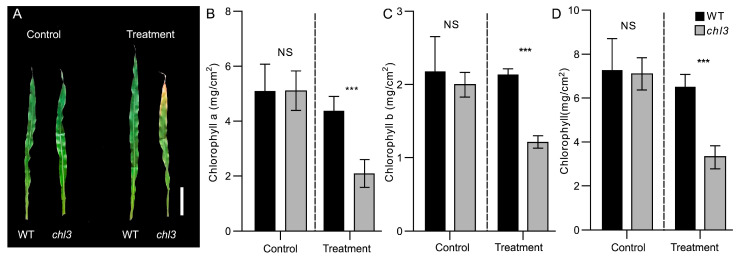
Cold treatment induces premature chlorosis in *chl3* leaves. (**A**) Phenotypes of mature leaves from *chl3* and wild-type (WT) plants grown under normal temperature conditions and after recovery from low-temperature exposure. Scale bar, 5 cm. Control, plants growing at normal temperature; cold treatment, plants returned to normal temperature for 7 days after being maintained at 8 °C for 2 days. (**B**–**D**) Chlorophyll *a* content (**B**), chlorophyll *b* content (**C**), and total chlorophyll content (D) of mature leaves from *chl3* and WT plants grown under normal temperature conditions or after recovery from low-temperature exposure. NS, no significant difference between mutant and WT; ***, *p*-value < 0.001 between mutant and WT, *t*-test.

Previous studies indicated that the leaves from the *chl3* mutant only develop chlorosis after the five-leaf stage when growing at a normal temperature of 28 °C [23], but we also found that *chl3* seedlings in spring fields exhibit early chlorosis after experiencing low temperatures. To verify whether the chlorosis phenotype of *chl3* was induced by low temperatures, we placed *chl3* seedlings at 8 °C for 2 days and restored them to 28 °C. On the seventh day after the temperature returned to normal (also the ninth day after the start of cold treatment), *chl3* began to exhibit a chlorosis phenotype (Figure 1A and Figure S1A). In support of this observation, the contents of chlorophyll *a*, chlorophyll *b*, and total chlorophylls of *chl3* leaves decreased by 52.3%, 43.2%, and 49.3%, respectively, relative to WT levels (Figure 1B–D). However, *chl3* seedlings sown at the same time and kept growing at 28 °C did not show any abnormalities (Figure 1A and Figure S1B), and the chlorophyll content was not significantly different from the wild type under the same conditions (Figure 1B–D). These results indicate that a short period of cold treatment at the seedling stage induces premature chlorosis of *chl3* leaves, suggesting that ZmPHOH, whose function is missing in *chl3*, may be involved in the cold response of maize seedlings. In addition, the malondialdehyde (MDA) content and electrolyte leakage of *chl3* leaves were significantly higher than those of the WT following 2d of recovery from cold treatment, indicating that *chl3* is more sensitive to low temperature (Figure S2A,B). suggesting that ZmPHOH, whose function is missing in *chl3*, may be involved in the cold response of maize seedlings.

### 2.2. The chl3 Mutant Has a Diminished Capacity for Photosynthetic Recovery After Short-Term Low-Temperature Treatment

**Figure 2:**
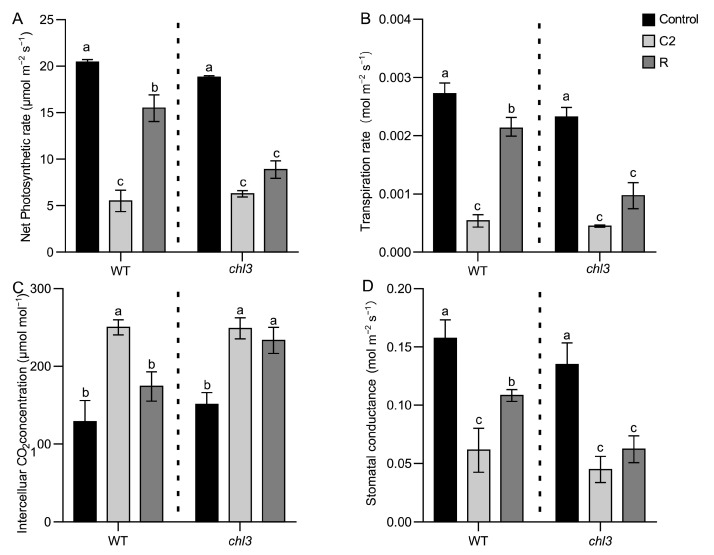
Changes in photosynthetic capacity during cold treatment and post-cold recovery in WT and *chl3* leaves. (**A**–**D**) Net photosynthetic rate (**A**), transpiration rate (**B**), intercellular CO_2_ concentration (**C**), and stomatal conductance (**D**) of WT and *chl3* leaves during cold treatment and after recovery from low-temperature exposure. Different lowercase letters indicate significant differences. WT, wild type. Control, before the start of cold treatment; C2, 48 h into cold treatment; R, 48 h after return to normal growing temperature after cold treatment.

As low temperatures damage the photosynthetic system, we speculated that cold-induced damage to the photosynthetic system might not be repaired after the alleviation of the cold stress in the *chl3* mutant, which would lead to premature chlorosis. To test this hypothesis, we measured various photosynthetic characteristics at different time points: before cold exposure (control), at 24 h (C1) or 48 h (C2) into the 8 °C cold treatment, and at 48 h after returning to 28 °C (R). Before cold treatment, there was no significant difference in the net photosynthetic rate, transpiration rate, intercellular CO_2_ concentration, or stomatal conductance between WT and *chl3* seedlings (Figure 2A–D). After 48 h of cold treatment, the net photosynthetic rate, transpiration rate, and stomatal conductance of WT and *chl3* seedlings all significantly decreased, while the intercellular CO_2_ concentration significantly increased. Importantly, all values were still comparable in the WT and *chl3* (Figure 2A–D), indicating that a 48 h cold treatment equally damaged the photosynthetic apparatus of WT and *chl3*. At 48 h, after returning to normal growing conditions at 28 °C, the intercellular CO_2_ concentration of the WT seedlings had fully returned to their pre-cold treatment level, while the values for net photosynthetic rate, transpiration rate, and stomatal conductance partially did, reaching 75.8%, 78.2%, and 68.4% of their pretreatment levels, respectively. By contrast, in *chl3* seedlings, the net photosynthetic rate, transpiration rate, intercellular CO_2_ concentration, and stomatal conductance remained at the same lower level as that during exposure to cold (Figure 2A–D). Based on these results, we conclude that, compared to the WT, *chl3* seedlings have a weaker ability to repair the photosynthetic damage caused by exposure to low temperatures, which may underlie their premature chlorosis.

### 2.3. ZmPHOH Activity Is Elevated After Short-Term Exposure to Low Temperature

**Figure 3:**
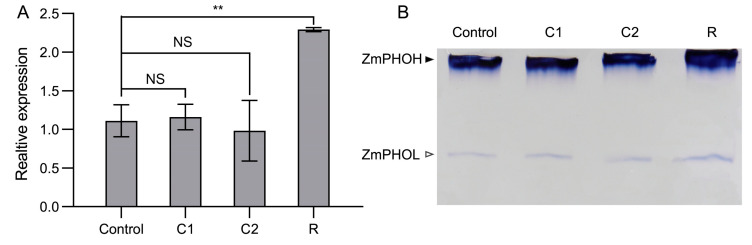
ZmPHOH plays a role in the post-cold recovery stage. (**A**) RT-qPCR analysis of *ZmPHOH* at different time points of cold treatment and recovery. Control, before the start of cold treatment; C1, 24 h into cold treatment; C2, 48 h into cold treatment; R, 48 h after recovery from low-temperature exposure. NS, no significant difference; **, *p*-value < 0.01, *t*-test. (**B**) In-gel ZmPHOH activity at different time points of cold treatment and recovery. Closed arrows mark the position of ZmPHOH (cytosolic starch phosphorylase), and the open arrow marks the position of ZmPHOL (plastid starch phosphorylase).

We wondered whether ZmPHOH was related to the cold response, as its loss of function in *chl3* seedlings resulted in premature leaf chlorosis. Accordingly, we measured *ZmPHOH* transcript levels and detected ZmPHOH enzyme activity in WT leaves at the control, C1, C2, and R time points. RT-qPCR analysis showed that *ZmPHOH* expression is not significantly different in seedlings exposed to cold treatment and control seedlings (*p*-value = 0.77 and 0.64, respectively); however, *ZmPHOH* transcript levels increased to twice that of the control during the post-cold recovery period (*p*-value = 0.0093) (Figure 3A). To analyze the influence of cold on ZmPHOH activity, we performed a native PAGE and activity staining assay. Specifically, we separated proteins under non-denaturing conditions, and then soaked the gel with soluble starch and G1P, which led to the formation of insoluble starch in the presence of a starch phosphorylase. After washing the gel and incubation with an iodine staining solution, we observed two bands in the gel. The upper band corresponded to ZmPHOH, which has a high affinity to glycogen and binds strongly to the immobilized highly branched polymer within the gel, while the lower band was for ZmPHOL, the plastid-localized isoform of starch phosphorylase with a lower affinity for glycogen. We observed no change in the activity of ZmPHOH during cold treatment, but noticed a marked increase during cold recovery Figure 3B and Figure S3, consistent with the RT-qPCR analysis. These results suggest that *ZmPHOH* is involved in the cold-stress response of maize seedlings and plays a role in post-cold recovery.

### 2.4. chl3 Leaves Maintain High Soluble Sugar Content Relative to the WT During Post-Cold Recovery

**Figure 4:**
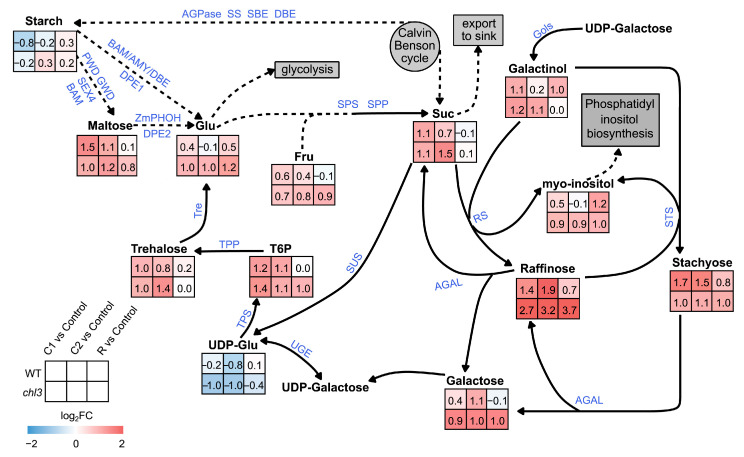
Changes of carbohydrate and carbohydrate derivatives contents in WT and *chl3* leaves during cold treatment and post-cold recovery. The numbers in each square indicate the Log_2_FC in the content of the corresponding metabolites in *chl3* and WT leaves during the cold treatment and post-cold recovery compared to pretreatment conditions. WT, wild type; FC, fold-change. C1, 24 h into cold treatment; C2, 48 h into cold treatment; R, 48 h into temperature recovery. Glu, glucose; Suc, sucrose; Fru, fructose; T6P, trehalose-6-phosphate. AGPase, ADP-glucose pyrophosphorylase; SS, starch synthase; SEB, starch branching enzyme; DBE, debranching enzyme; BAM, β-amylase; AMY, α-amylase; PWD, phosphoglucan water di-kinase; GWD, glucan water dikinase; DPE, disproportionating enzyme; SPS, sucrose phosphate synthase; SPP, sucrose phosphate phosphatase; SUS, sucrose synthase; TPP, trehalose-6-phosphate phosphatase; TPS, trehalose-6-phosphate synthetase; UGE, UDP-glucose 4-epimerase; AGAL, α-galactosidase; RS, raffinose synthase; STS; stachyose synthase; Gols, galactinol synthase.

To investigate the role of ZmPHOH in carbohydrate metabolism during cold treatment and the subsequent recovery stage, we measured the contents of various carbohydrates in WT and *chl3* leaves at the control, C1, C2, and R time points by gas chromatography–mass spectrometry. After 24 h of cold treatment, the starch content of WT and *chl3* leaves decreased compared to that in the control samples not exposed to cold. More precisely, the starch content decreased by 42.6% in WT leaves and by 12.9% in *chl3* leaves. Conversely, the contents of soluble sugars, such as maltose, glucose, sucrose, fructose, raffinose, stachyose, and galactose, all significantly increased in both WT and *chl3* leaves (Figure 4, Table S1), suggesting that cold treatment promotes the conversion of starch to soluble sugars. After 48 h of cold treatment, the starch levels in WT and *chl3* leaves began to rise, almost returning to the control levels, but the contents of the soluble sugars maltose, sucrose, fructose, stachyose, raffinose, and galactose remained at high levels. Of all soluble sugars, raffinose accumulated to the highest levels, increasing 3.7 times in WT leaves and 9.2 times in *chl3* leaves relative to their respective controls (Figure 4), indicating that soluble sugars, especially RFOs, play an important role in resisting cold stress [24,25,26]. After 48 h of recovery from cold treatment, the starch contents of WT and *chl3* leaves increased slightly compared to the control. However, the contents of the soluble sugars maltose, glucose, sucrose, fructose, raffinose, stachyose, and galactose decreased significantly in WT leaves after transfer back to the normal growing temperature; the contents of maltose, sucrose, fructose, and galactose all returned to the same low levels seen in the leaves of control seedlings, while the levels of raffinose and stachyose remained relatively high (Figure 4). In *chl3* leaves, the contents of maltose, glucose, fructose, raffinose, stachyose, and galactose also remained high after the seedlings were transferred from the cold treatment back to the normal growing temperature (Figure 4), indicating that the loss of ZmPHOH function in *chl3* leaves compromised the catabolism of soluble sugars seen upon cold treatment in the WT, revealing that ZmPHOH plays an important role in the catabolism and metabolism of soluble sugars during post-cold recovery.

### 2.5. Differentially Expressed Genes in WT and chl3 Leaves During and After Cold Treatment Have Similar Functions

To compare the global changes in gene expression in the WT and *chl3* during and after cold treatment, we performed a comparative transcriptome analysis using total RNA extracted from the WT and *chl3* leaves at the control, C2, and R time points for transcriptome deep sequencing (RNA-seq). A principal component analysis (PCA) showed that all three biological replicates for each genotype and time point cluster together in the PCA plot, indicative of high reproducibility (Figure S4A).

**Figure 5:**
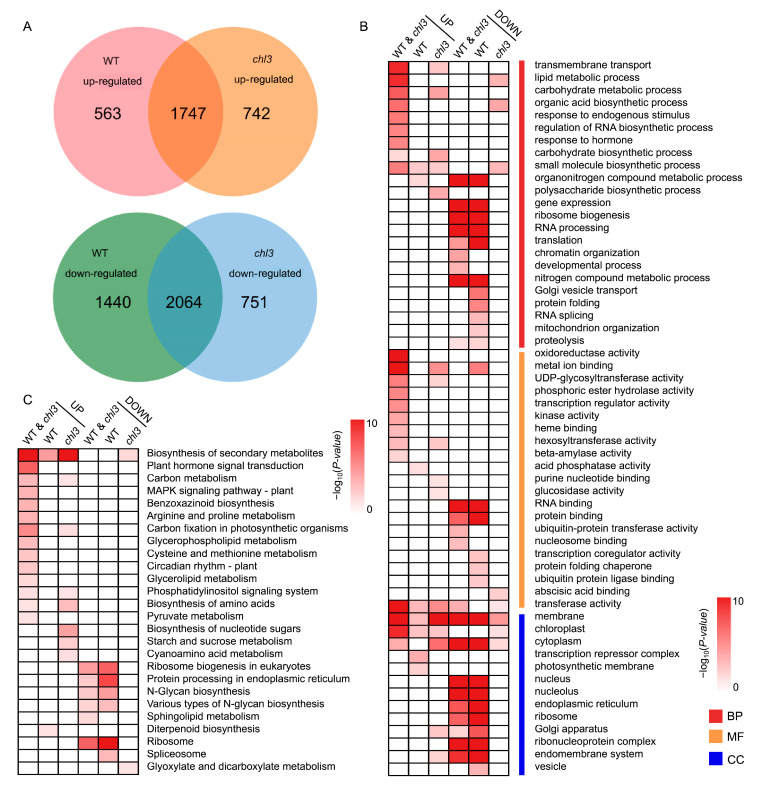
Transcriptome analysis of WT and *chl3* leaves after cold treatment. (**A**) Venn diagrams showing the extent of overlap between the number of differentially expressed genes (DEGs) in the wild type (WT) and *chl3*. (**B**) Gene Ontology term enrichment analysis of DEGs for the indicated pairwise comparisons. BP, biological process; MF, molecular function; CC, cellular component. (**C**) Kyoto Encyclopedia of Genes and Genomes pathway enrichment analysis of DEGs for the indicated pairwise comparisons.

We identified 1747 upregulated genes and 2064 downregulated genes shared by the WT and *chl3* after 48 h of cold treatment when compared to the control samples maintained at 28 °C (Figure 5A and Figure S4B). These shared upregulated genes were significantly enriched in Gene Ontology (GO) terms and Kyoto Encyclopedia of Genes and Genomes (KEGG) pathways related to stress response and substance metabolism, including response to endogenous stimulus (GO: 0009719, *p*-value = 6.68 × 10^−7^), lipid metabolic process (GO: 0006629, *p*-value = 7.12 × 10^−10^), carbohydrate biosynthetic process (GO: 001605, *p*-value = 2.08 × 10^−2^), biosynthesis of secondary metabolites (KEGG orthology [ko]: 01110, *p*-value = 2.51 × 10^−21^), and carbon metabolism (ko: 00710, *p*-value = 5.11 × 10^−6^). The genes specifically upregulated in the WT were enriched in similar GO and KEGG terms as the above co-upregulated genes, while the genes specifically upregulated in *chl3* were enriched for GO terms and KEGG pathways related to carbohydrate metabolism (Figure 5B,C). The downregulated genes shared by WT and *chl3* were significantly enriched in GO terms and KEGG pathways related to transcription and translation, such as ribosome biosynthesis (GO: 0010467, *p*-value = 1.28 × 10^−15^), RNA processing (GO: 0006396, *p*-value = 6.39 × 10^−12^), translation (GO: 0006412, *p*-value = 2.06 × 10^−5^), and ribosome biogenesis in eukaryotes (ko: 03008, *p*-value = 3.27 × 10^−5^). The GO and KEGG terms significantly enriched among the downregulated genes, specifically in the WT, were also related to transcription and translation, while the genes downregulated in *chl3* only were significantly enriched for GO terms and KEGG pathways mainly related to substance metabolism (Figure 5B,C). We conclude that following 48 h of exposure to cold, WT and *chl3* leaves initiated similar stress responses by adjusting their metabolism and avoided dissipating excess energy by decreasing the rate of RNA transcription and protein translation. However, compared to the WT, the loss of ZmPHOH function in *chl3* led to differences in carbohydrate metabolism.

**Figure 6:**
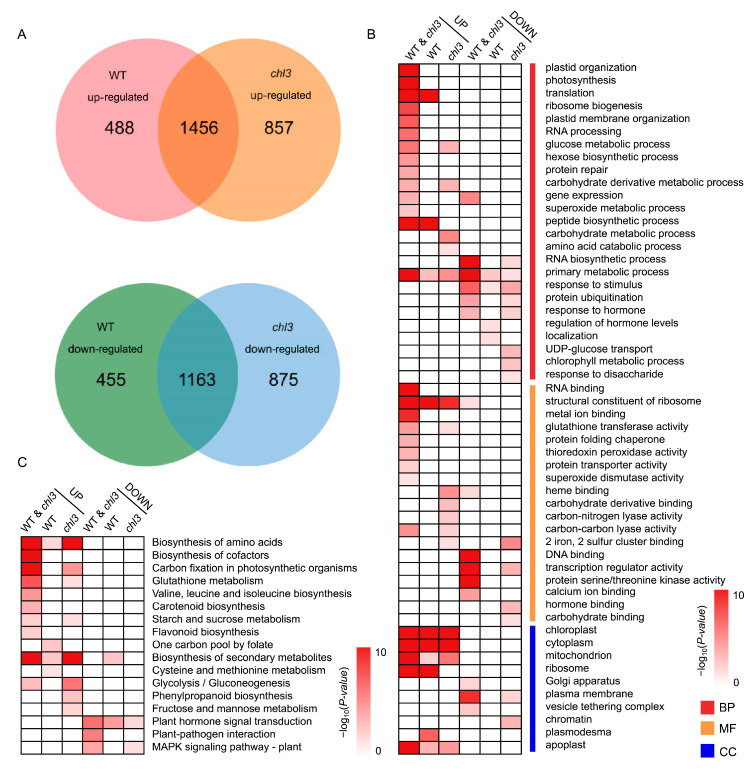
Transcriptome analysis of WT and *chl3* leaves during the recovery stage after cold treatment. (**A**) Venn diagrams showing the extent of overlap in DEGs between the WT and *chl3*. (**B**) Gene Ontology enrichment analysis of DEGs for the indicated pairwise comparisons. BP, biological process; MF, molecular function; CC, cellular component. (**C**) Kyoto Encyclopedia of Genes and Genomes pathway enrichment analysis of DEGs for the indicated pairwise comparisons.

After 48 h of return to normal growing conditions, we identified 1456 upregulated genes and 1163 downregulated genes shared by the WT and *chl3* (Figure 6A and Figure S3B). These shared upregulated genes were significantly enriched in GO terms and KEGG pathways related to photosynthesis, transcription and translation processes, and carbohydrate metabolism, such as photosynthesis (GO: 0015979, *p*-value = 3.47 × 10^−32^), RNA binding (GO: 0003723, *p*-value = 7.34 × 10^−24^), translation (GO: 0006412, *p*-value = 2.34 × 10^−20^), carbohydrate derivative metabolic process (GO: 1901135, *p*-value = 1.62 × 10^−4^), and starch and sucrose metabolism (ko: 00500, *p*-value = 5.34 × 10^−3^). The significantly enriched GO terms and KEGG pathways among upregulated genes specifically in the WT were similar to those for the above co-upregulated genes, while the GO terms and KEGG pathways significantly enriched among the upregulated genes specific to *chl3* were related to carbohydrate metabolism (Figure 6B,C). The downregulated genes shared by the WT and *chl3* were significantly enriched in GO terms and KEGG pathways related to stress response and gene expression regulation, including response to stimulus (GO: 0050896, *p*-value = 8.11 × 10^−8^), gene expression (GO: 0010467, *p*-value = 3.11 × 10^−6^), plant hormone signal transduction (ko: 04075, *p*-value = 2.20 × 10^−7^), and mitogen-activated protein kinase signaling pathway (ko: 04016, *p*-value = 7.43 × 10^−5^). The GO terms and KEGG pathways significantly enriched among genes downregulated specifically in the WT or *chl3* were similar to those identified for the co-downregulated genes; in addition, the GO terms and KEGG pathways significantly enriched among the genes specifically downregulated in *chl3* were related to the metabolism of carbohydrates and their derivatives (Figure 6B,C). We conclude that the stress response transcriptome changes in WT and *chl3* leaves are terminated after the end of the cold treatment and return to normal growing conditions, which initiates the gradual recovery of previously cold-inhibited biological processes such as transcription and translation. However, the clearest difference between the WT and *chl3* lies in their distinct metabolic patterns for carbohydrates, indicating that the loss of ZmPHOH affects the recovery of maize from cold stress by disrupting carbohydrate metabolism.

### 2.6. Alterations in Carbohydrate Metabolism and Photosynthesis-Related Pathways in chl3 Leaves During and After Cold Treatment

**Figure 7:**
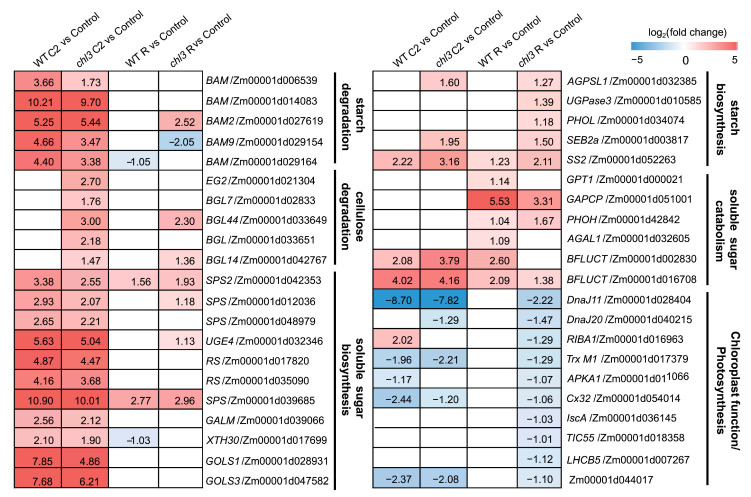
Carbohydrate metabolism and photosynthesis-related DEGs during cold treatment and post-cold recovery in the WT and *chl3.* Control, before the start of cold treatment; C2, 48 h into cold treatment; R, 48 h after recovery from low-temperature exposure. WT, wild type; DEGs, differentially expressed genes.

Previous studies have shown that the expression of genes related to starch degradation and soluble sugar biosynthesis was upregulated during cold treatment, while the expression of genes related to soluble sugar catabolism was activated after resumption of normal growing conditions after cold exposure [11,12,13,14,15,16,27]. We therefore investigated the changes in carbohydrate metabolism in *chl3* leaves during the cold response by analyzing the expression levels of the relevant genes. During cold treatment, several key genes involved in starch degradation and soluble sugar biosynthesis were significantly upregulated in both the WT and *chl3*, such as *BAM*, *Sucrose Phosphate Synthase* (*SPS*), *Raffinose Synthase* (*RS*), and *Stachyose Synthase* (*STS*) genes [28], while some genes involved in cellulose degradation were upregulated only in *chl3*, such as *Endoglucanase* (*EG*) and *β-Glucosidase* (*BGL*) genes [29] (Figure 7). During post-cold recovery, the expression levels of some key genes related to soluble sugar degradation were upregulated specifically in WT leaves, such as α-Galactosidase (*AGAL*) and *β-fructofuranosidase* (*BFLUCT*) genes [28] (Figure 7). These results may help explain why the levels of soluble sugars in *chl3* leaves remained at high levels after cold treatment.

To investigate whether the increased contents of soluble sugars in *chl3* leaves caused a feedback inhibition of photosynthetic gene expression, we assessed the expression levels of related genes in the WT and *chl3*. After cold treatment and return to the normal growing temperature, the expression levels of genes encoding proteins such as light harvesting complex B5, chloroplast thioredoxin M1, and chloroplast chaperone protein DnaJ returned to pre-cold exposure levels in WT leaves but were significantly downregulated in *chl3* leaves (Figure 7). In summary, excessive levels of soluble sugars appear to inhibit the expression of photosynthetic genes in *chl3* leaves during post-cold recovery, making it difficult to restore the photosynthetic capacity of *chl3*, which may ultimately lead to premature leaf chlorosis.

## 3. Discussion

### 3.1. The Decreased Cold Tolerance of chl3 Is Caused by Abnormal Carbohydrate Metabolism

WT and *chl3* seedlings exhibit a similar appearance during the seedling stage when grown under the normal temperature condition of 28 °C. Starting around the five-leaf stage and until the end of the growth period, mature *chl3* leaves begin to turn chlorotic. The *chl3* mutant is defective in the starch phosphorylase ZmPHOH, leading to the gradual accumulation of carbohydrates in its leaves [23]. In this study, we determined that when exposing *chl3* seedlings to short-term cold treatment (48 h at 8 °C) during the seedling stage, the young *chl3* leaves turned chlorotic as would the mature leaves of the mutant when grown under the normal condition of 28 °C (Figure 1A and Figure S1A,B), and the photosynthetic capacity of the mutant was inhibited before chlorosis became apparent (Figure 2A), suggesting that *chl3* is less cold tolerant than the WT.

**Figure 8:**
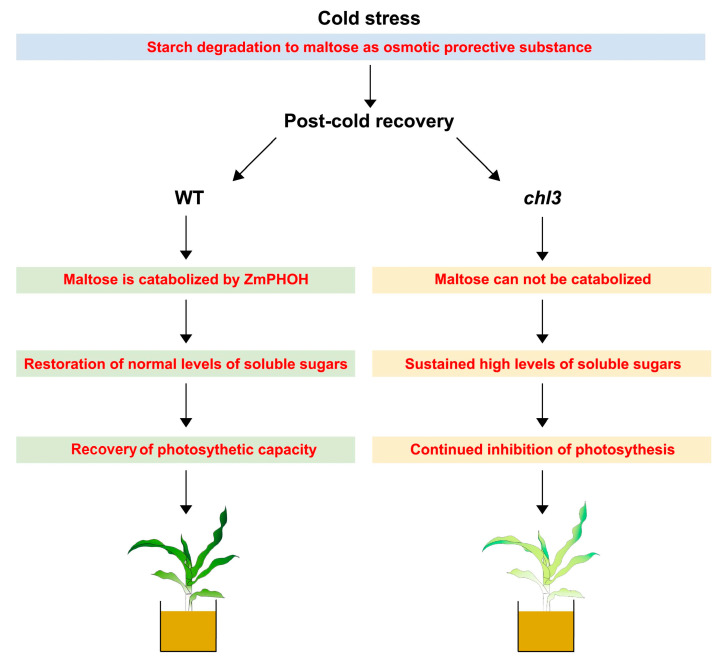
A proposed model of *ZmPHOH* affecting carbohydrate metabolism during post-cold recovery and leading to changes in cold tolerance. The loss of ZmPHOH function in *chl3* leaves resulted in the inability to effectively decompose maltose when the growth temperature returned to normal, thereby disrupting the metabolism of other sugars and maintaining the content of soluble sugars at a high level, which in turn suppressed the expression of photosynthesis genes and impeded the restoration of photosynthesis capacity, resulting in chlorotic leaves. WT, wild-type.

To investigate whether the decreased tolerance to cold seen in the *chl3* mutant was associated with altered carbohydrate metabolism, we measured its carbohydrate contents at different growth stages during and after cold treatment. We established that the starch content of both WT and *chl3* leaves decreased before increasing under cold treatment (Figure 4), suggesting that low temperature caused a reprogramming of carbohydrate metabolism, with an improved turnover rate of transitory starch, thus accelerating starch biosynthesis. We also observed an accumulation of soluble sugars in both WT and *chl3* leaves during cold treatment (Figure 4), indicating that soluble sugars play an important role in resisting cold stress. In summary, the carbohydrate metabolism patterns in WT and *chl3* leaves were rather similar and consistent with previous reports of sugar metabolism during cold treatment in different species [11,14,15,16,30,31]. However, large differences emerged in carbohydrate metabolism between the WT and *chl3* during the post-cold recovery period. The soluble sugars that had accumulated under cold treatment became rapidly catabolized, largely returning to pre-cold stress levels in WT leaves upon resumption of normal growing conditions at 28 °C; by contrast, the accumulated soluble sugars in *chl3* leaves persisted for at least 48 h after return to the normal growing temperature (Figure 4). Excess accumulation of soluble sugars in leaves inhibits the expression of photosynthetic genes such as *LHCB* and *Trx* [17,32,33], making it difficult to fully restore photosynthesis efficiency, and causes an overflow effect, leading to the excessive accumulation of starch in the leaves at later stages [34,35,36] and ultimately resulting in leaf chlorosis. Therefore, we attribute the difficulty in relieving photosynthetic inhibition and counteracting the premature leaf chlorosis seen in *chl3* after cold treatment to the accelerated accumulation of carbohydrates as a result of the low temperature, followed by a defect in rapid carbohydrate catabolism after the normal growing temperature was restored (Figure 8).

### 3.2. ZmPHOH Promotes Soluble Sugars Catabolism During Post-Cold Recovery

The substrate for starch phosphorylase 2 is soluble heteroglycan (SHG), the receptor for glucose residues during maltose degradation. Firstly, maltose releases a free glucose in the presence of disproportionating isozyme2 (DPE2), and the remaining glucose residue is bound to SHG. Subsequently, the glucose residues in SHG are phosphorylated by starch phosphorylase 2 to generate G1P. Starch phosphorylase 2 ensures the circulation of glucose residues in SHG, and therefore its activity has a strong influence on the efficiency of maltose degradation [23,37,38]. Under normal conditions, maltose is only an intermediate product of transitory starch remobilization at night and is quickly converted to sucrose [37,38]. Under cold conditions, maltose plays an important role as an osmoprotectant for maintaining protein stability, protecting cell membranes and electron transport chains and clearing ROS; maltose is not completely consumed as quickly as under a normal growing temperature [10,12,39]. Therefore, the inability to degrade maltose in a timely manner due to the inactivation of ZmPHOH did not have severe consequences during cold exposure. Importantly, when the seedlings were returned to a normal growing temperature, the activity of enzymes related to soluble sugar catabolism increased to catabolize the previously accumulated soluble sugars [5,22], at which point the importance of ZmPHOH becomes apparent. To determine the response of ZmPHOH to low temperatures, RT-qPCR and native PAGE analyses were performed at different time points in the cold treatment, showing that *ZmPHOH* transcript levels and ZmPHOH enzyme activity in WT leaves significantly increased during the post-cold recovery period (Figure 2A,B). Notably, native PAGE also showed that ZmPHOL, the isoform of ZmPHOH, exhibited increased activity, which may be related to its elevated expression (Figure 7). In wild-type leaves, the highly active ZmPHOH ensures that excess maltose is converted into glucose and G1P, which is channeled to other metabolic pathways. The loss of ZmPHOH function in *chl3* leaves leads to an inability to catabolize maltose efficiently after the growing temperature returned to normal, thereby disrupting the metabolism of other sugars and maintaining high levels of soluble sugars. These high contents of soluble sugars in turn inhibit the expression of photosynthetic genes, hindering the recovery of photosynthetic ability and adversely affecting the repair caused by cold exposure. Since the eventual effect of cold stress on photosynthesis is not only a result of the extent of the damage to the photosynthetic apparatus, but also depends on the capacity for recovery after the damage has taken place, we speculate that ZmPHOH affects cold tolerance in maize by participating in the catabolism of soluble sugars during post-cold recovery (Figure 8).

There have been many reports on optimizing carbohydrate metabolism to help plants withstand cold stress. Enzymes functioning upstream of starch phosphorylase 2, such as GWD1 and BAM7 or BAM8, modulate cold tolerance in Arabidopsis, albeit by facilitating the degradation of transitory starch to soluble sugars [11,12,39]. Thus, these enzymes can be considered as an immediate line of protection against cold stress. We showed here that ZmPHOH acts during the recovery stage to prevent sustained high levels of soluble sugars from inhibiting plant photosynthetic capacity and impairing subsequent growth and development. The main function of ZmPHOH is thus to enable plants to adjust their metabolism in response to cold stress and readjust when returning to more clement growth conditions. Starch phosphorylase 2 may therefore play a unique role in regulating plant cold tolerance.

### 3.3. Multiple Functions for Starch Phosphorylase 2 in Plant Growth and Development

Carbohydrates are not only energetic and structural substances, but also serve as signaling molecules to regulate plant growth and development. Therefore, enzymes related to carbohydrate metabolism often play multiple roles in plant growth and development. Starch phosphorylase is a key enzyme in glucan metabolism by catalyzing the reversible transfer of glucosyl units from G1P to the nonreducing end of α-1,4-glucan chains with the release of phosphate. Two distinct forms of starch phosphorylase have been consistently observed: starch phosphorylase 1 and starch phosphorylase 2. The role of starch phosphorylase 1 is complex, and there is no clear conclusion as to which metabolic pathway it is involved in [40]. By contrast, studies related to starch phosphorylase 2 are sufficiently advanced to identify its metabolic pathway and diverse biological functions in different species.

Starch accounts for more than 40% of the total carbohydrates present in maize leaves; however, starch is not a major carbohydrate in rice leaves (less than 1%), but it is in rice pollen [41,42]. The loss of ZmPHOH function in the *chl3* mutant hindered the remobilization of transitory starch in maize and restricted the supply of material and energy to storage organs, ultimately decreasing biomass and yield [23]. The mutation of *OsPHO2* causes lower fertility due to the inability to metabolize starch properly in rice pollen but does not otherwise affect plant growth [43]. These results indicate that since the site of starch distribution varies from species to species, starch phosphorylase 2 likely functions in different tissues, depending on the plant species, to regulate either vegetative growth or reproductive growth. Moreover, starch phosphorylase 2 also regulates plant stress tolerance.

In this study, we revealed that ZmPHOH participates in the repair of photosynthetic damage imposed by cold exposure by catabolizing soluble sugars during the recovery stage, thus affecting the acclimation of maize seedlings to low temperature. *ALPHA-GLUCAN PHOSPHORYLASE 2* (*AtPHS2*), the homologue of *ZmPHOH* in Arabidopsis was shown to help plants cope with an imbalance in carbohydrate levels at different developmental stages [44], suggesting that starch phosphorylase 2 may help resist various abiotic stresses. In summary, starch phosphorylase 2 has multiple functions in plant growth and development and may be an important target for genetic improvement in the future; for example, its manipulation may help synergize yield and cold tolerance in maize.

## 4. Materials and Methods

### 4.1. Plant Materials and Treatments

**Figure 9:**
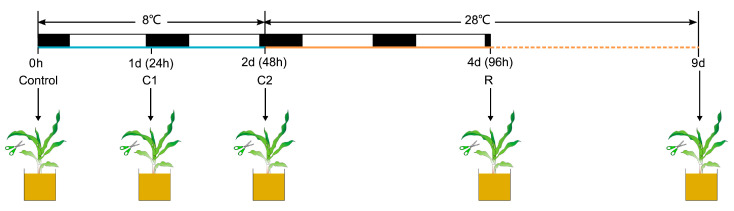
Experimental design for cold treatment and post-cold recovery. The blue line represents 8 °C. The orange line represents 28 °C, with the dashed line indicating the continuation of the previous photoperiod at 28 °C.

The *chl3* mutant used in this study is in the PH6WC maize (*Zea mays*) inbred line background and was described by Qin et al. (2022). Seeds for the wild-type PH6WC and *chl3* were germinated on tissue paper soaked with distilled water in the dark at room temperature for 2 days. When the roots were about 1.5 cm in length, uniform seedlings were transplanted into pots containing soil. Plants were grown in a growth chamber at 28 °C for 25 days before being transferred to 8 °C for cold treatment for 2 days, then returned to 28 °C. All growth chambers were controlled at 65% humidity and under a 14/10 h photoperiod with 40 μmol m^−2^ s^−1^ photosynthetically active radiation during the light part. The start of the cold treatment was 1 h after lights off so interference from the light signal can be avoided (Figure 9). All experiments were performed at the same time of the light cycle and were repeated at least three times.

The different sampling time points are represented as follows: control indicates before the start of cold treatment, C1 (Cold treatment1) indicates 24 h after cold treatment, C2 (Cold treatment2) indicates 48 h after cold treatment, and R (Recovery) indicates 48 h after temperature recovery (Figure 9). WT leaves were collected at the time points of control, C1, C2, and R to analyze the expression level of *ZmPHOH* and starch phosphorylase activity. WT and *chl3* leaves were harvested at the control, C2, and R time points for total RNA extraction and transcriptome analysis.

### 4.2. Determination of Chlorophyll Content

Following a 7-day recovery period after cold treatment, wild-type (WT) leaves, and the chlorotic sections of *chl3* leaves were collected for chlorophyll content analysis (Figure 9). Six biological replicates were established for each sample type. The collected leaf tissues were carefully cut into 1-cm^2^ fragments and subsequently immersed in 10 mL of 80% (*v*/*v*) acetone solution. The extraction process was conducted in complete darkness for 24 h to prevent chlorophyll degradation. The absorbance of the resulting extracts was measured at two specific wavelengths (645 nm and 663 nm) using a UV-1800 spectrophotometer (MAPADA, Shanghai, China). Chlorophyll concentrations were determined using the following established formula:

C_Chl.*a*_ = 12.27 × OD_663_ − 2.69 × OD_645_; C_Chl.*b*_ = 22.9 × OD_645_ − 4.68 × OD_663_

### 4.3. Quantification of Malondialdehyde Content and Electrolyte Leakage

The content of malondialdehyde (MDA) and the extent of electrolyte leakage were measured in the WT and the *chl3* mutant after 2 days of cold treatment. The MDA content was quantified using Plant Malondialdehyde assay kit (Nanjing Jiancheng Bioengineering Institute, Nanjing, China) based on the manufacturer’s protocols. For electrolyte leakage, leaves harvested from cold-treated plants were washed with Milli-Q (MQ) water before being incubated in a 50 mL tube filled with 30 mL MQ water for 12 h, after which the initial ion conductivity_1_ (C_1_) was measured using a conductivity meter (Lutron Electronics Co., Inc., Coopersburg, PA, USA). All tubes were then autoclaved at 121 °C for 10 min, and the total conductivity was measured (C_2_) once the samples reached 20–25 °C. The electrolyte leakage rate (EL) was calculated using the following formula:

EL = C_1_/C_2_ × 100% (1)

### 4.4. Gas Exchange Measurements

Gas exchange and photosynthesis measurements were performed using a portable infrared gas exchange system (LI-6800, LI-COR Inc., Lincoln, NE, USA) on the youngest fully expanded leaves. Net photosynthesis (Anet, μmol CO_2_ m^−2^ s^−1^), transpiration (E, mol H_2_O m^−2^ s^−1^), intercellular CO_2_ concentration (Ci, μmol CO_2_ mol^−1^), and stomatal conductance (gs, mol H_2_O m^−2^ s^−1^) were measured at a photon flux density of 1500 μmol m^−2^ s^−1^ with a CO_2_ concentration adjusted to 400 μmol mol^−1^ using a CO_2_ mixer. Each experiment was repeated six times.

### 4.5. RNA Extraction and RT-qPCR

Total RNA was isolated from leaf samples collected at designated time points using TRIzol reagent (1 mL per sample; Thermo Fisher Scientific, Waltham, MA, USA), following the manufacturer’s protocol. Following isopropanol precipitation, the RNA pellets were resuspended in 50 µL of RNase-free water and subsequently treated with DNase I (RNase-free) to eliminate potential genomic DNA contamination. For cDNA synthesis, 1 μg of total RNA from each sample was reverse transcribed in a 20-μL reaction volume using the HiScript II 1st Strand cDNA Synthesis Kit (Vazyme, Nanjing, China), in accordance with the manufacturer’s instructions. Quantitative PCR (qPCR) was performed using the SYBR Green master mix (Bio-Rad, Hercules, CA, USA) according to the manufacturer’s recommendations. Each sample was analyzed in triplicate using a Bio-Rad CFX96 Touch Real-Time PCR detection system, with *ZmActin1* serving as the internal reference gene (Table S2). Relative mRNA expression levels were determined using the comparative 2^−ΔΔCt^ method [45], with three independent biological replicates analyzed for each time point.

### 4.6. Native PAGE

For soluble protein extraction, leaf samples (3 g) were rapidly frozen in liquid nitrogen and homogenized to a fine powder using a pre-chilled mortar and pestle. The powdered tissue was resuspended in 10 mL of ice-cold protein extraction buffer (100 mM HEPES-NaOH, pH 7.5, 1 mM EDTA, 5 mM dithioerythritol, 0.5 mM phenylmethylsulfonyl fluoride, and 10% [*v*/*v*] glycerol). The homogenate was centrifuged at 20,000× *g* for 12 min at 4 °C, and the resulting supernatant was collected as the crude protein extract.

Glycogen-containing native PAGE was performed following established protocols [46]. The separation gel (7.5% [*w*/*v*]) was prepared with 0.11 M Tris-HCl (pH 7.2) and 0.2% (*w*/*v*) glycogen, followed by overnight dialysis. Electrophoresis was conducted at 4 °C using 38 mM Tris-glycine (pH 8.5) as the running buffer, with a constant voltage of 180 V. Each lane was loaded with 8 mg of soluble protein. Following electrophoresis, the gel was equilibrated in 100 mM citrate-NaOH buffer (pH 6.5) containing 0.05% (*w*/*v*) soluble starch for 30 min at room temperature. The gel was then incubated overnight at room temperature in reaction buffer consisting of 100 mM citrate-NaOH (pH 6.5), 0.05% (*w*/*v*) soluble starch, and 20 mM glucose-1-phosphate (G1P). Finally, the gel was stained with iodine solution (100 mM citrate-NaOH, pH 6.5, containing 0.67% [*w*/*v*] I₂ and 3.33% [*w*/*v*] KI) and destained in deionized water until clear background was achieved.

### 4.7. Soluble Sugar and Starch Quantification

The harvested leaves were finely ground into powder using liquid nitrogen, with six biological replicates prepared for each sample. Each sample was then treated with 1400 μL of methanol, which included 2 μL of c14-d27 as an internal standard. The mixtures were transferred to glass tubes and subjected to sonication for 30 min at 1000 rpm, with intermittent pauses every 5 min. Following sonication, the samples were centrifuged at 4000 rpm for 5 min to separate the supernatant from the pellet.

The supernatant was carefully transferred to an autosampler vial equipped with an insert and subsequently dried under a gentle stream of nitrogen to facilitate the extraction of soluble sugars. For further processing, 40 μL of methoxyamine hydrochloride was added to the residual pellet, and the mixture was incubated at 37 °C for 1 h. Subsequently, 80 μL of N,O-bis(trimethylsilyl)trifluoroacetamide (BSTFA), containing 1% (*w*/*v*) trimethylchlorosilane (TMCS), was introduced, and the samples were incubated at 60 °C for an additional hour. The soluble sugar content was then quantified using gas chromatography–mass spectrometry (GC-MS; model 8890-5977B, Agilent Inc., Santa Clara, CA, USA).

For starch extraction, 330 μL of dimethyl sulfoxide (DMSO) was added to the pellet, and the mixture was heated in a boiling water bath for 5 min to gelatinize the starch. A 50 μL aliquot of the gelatinized starch was then diluted with 950 μL of 100 mM sodium acetate buffer (pH 5.0). This was followed by the addition of 100 μL of α-amylase working solution (~30 U) and incubation in a boiling water bath for 15 min, after which the samples were transferred to a 50 °C water bath for 3 min. Subsequently, 20 μL of amyloglucosidase (~66 U) was added to each sample, and the mixtures were incubated at 50 °C for 1 h. The samples were then centrifuged at maximum speed for 5 min at room temperature, and the resulting supernatant was transferred to a new 1.5-mL centrifuge tube. The glucose content in the supernatant, which served as a proxy for starch content, was measured using a glucose (HK) assay kit (Sigma, Saint Louis, MO, USA).

### 4.8. RNA-Seq Analysis

Total RNA was isolated from the collected leaf samples. Sequencing libraries were prepared following the standard Illumina protocol and subsequently sequenced on an Illumina NovaSeq platform (Novogene, Beijing, China). The raw sequencing reads were quality-trimmed using Trimmomatic (version 0.33) and aligned to the B73 RefGen_v4.34 reference genome using HISAT2 (version 2.1.0). Transcript assembly and gene expression quantification were performed using StringTie (version 1.3.3b) [47]. Gene-level read counts were obtained using HTSeq (version 0.6.1). Differential expression analysis was conducted using DEseq2 (Version:1.45.1, http://www.bioconductor.org/packages/devel/bioc/html/DESeq2.html accessed on 8 March 2023), with significant differentially expressed genes (DEGs) identified based on a threshold of *p* < 0.01 and an absolute log2[fold-change] > 1. Functional annotation of DEGs was performed through integrated Gene Ontology (GO) term and Kyoto Encyclopedia of Genes and Genomes (KEGG) pathway enrichment analyses using the “g:Profiler” tool (http://biit.cs.ut.ee/gprofiler/ accessed on 6 June 2011).

### 4.9. Statistical Analysis

All experiments included at least three biological repeats and are shown as means ± standard deviation. The data for each variety under different treatment conditions were independently calculated. One-way analysis of variance (ANOVA) and two-way ANOVA were performed to assess the significance of differences between groups using GraphPad Prism 8.3.0 software. The criterion of *p* < 0.05 was used as the statistically significant threshold in this study.

## 5. Conclusions

Low temperature exerts detrimental effects on the photosynthetic system of maize, making it crucial to promote the recovery of photosynthetic capacity following cold exposure. ZmPHOH participates in the decomposition of low temperature-induced excessive soluble sugars during post-cold recovery, which alleviates the repression of photosynthetic gene expression and facilitates the restoration of photosynthetic capacity, thereby serving as a potential target for genetic improvement of cold tolerance in maize. Given the multifunctional nature of starch phosphorylase 2, future enhancement of ZmPHOH enzymatic activity through gene editing and other biotechnological approaches holds promise for synergistically improving both yield and cold tolerance in maize.

## Data Availability

The original contributions presented in this study are included in the article. Further inquiries can be directed to the corresponding author.

## Supplementary Materials

The following supporting information can be downloaded at: https://www.mdpi.com/article/10.3390/ijms26041727/s1.
