# Supplementary material for: A Starch Phosphorylase, ZmPHOH, Improves Photosynthetic Recovery from Short-Term Cold Exposure in Maize
Source: Int J Mol Sci. 2025 Feb 18;26(4):1727. doi: 10.3390/ijms26041727 (PMC11855087; doi:10.3390/ijms26041727)
Supplement: Supplementary file 1 [file ijms-26-01727-s001.zip › ijms-3441385-supplementary.pdf]

SUPPLEMENTARY MATERIAL

There are two supplementary tables and four supplementary figures, including Supplementary Table S1,S2 and Supplementary Figure S1-S4.

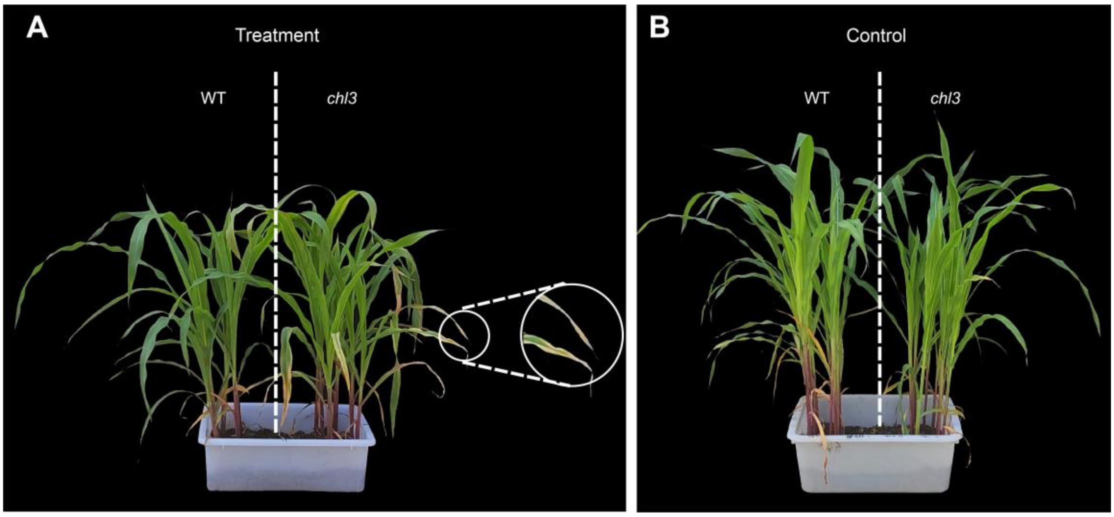

Figure S1 Low temperature induces the production of a chlorosis in *chl3* leaves

(A-B)Phenotypes of *chl3* and wild-type plants under treatment (A) and control (B) conditions. Control, plants grow at normal temperatures; Treatment, plants returned to normal temperature for 7 days after being treated at 8 °C for 2 days. WT, wild-type.

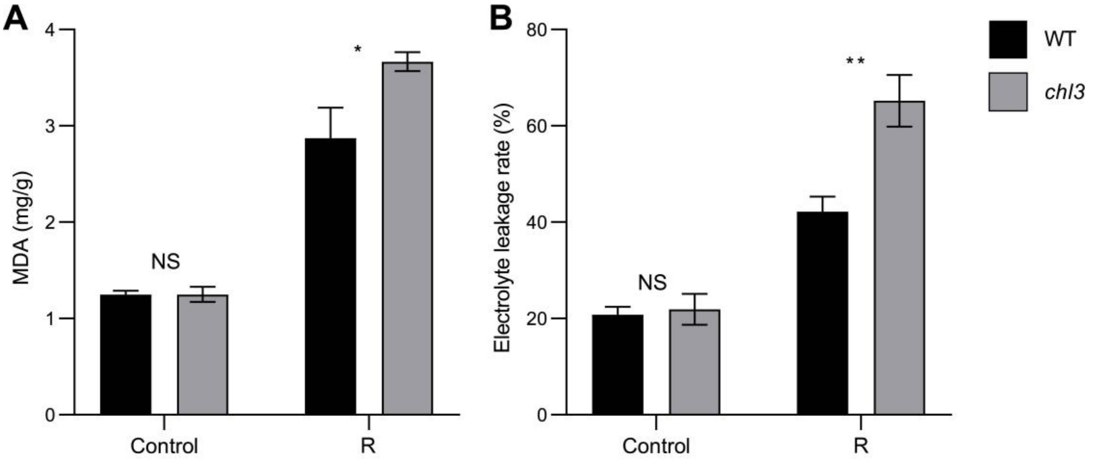

Figure S2 *chl3* is more sensitive to low temperatures

(A-B) MDA content (A) and electrolyte leakage rate (B) of *chl3* and wild-type leaves. Control, plants grow at normal temperatures; R, 48 h after temperature recovery. WT, wild-type. (NS：no significant difference, “*”：*P-value* < 0.05，“**”：*P-value* < 0.01，t-test)

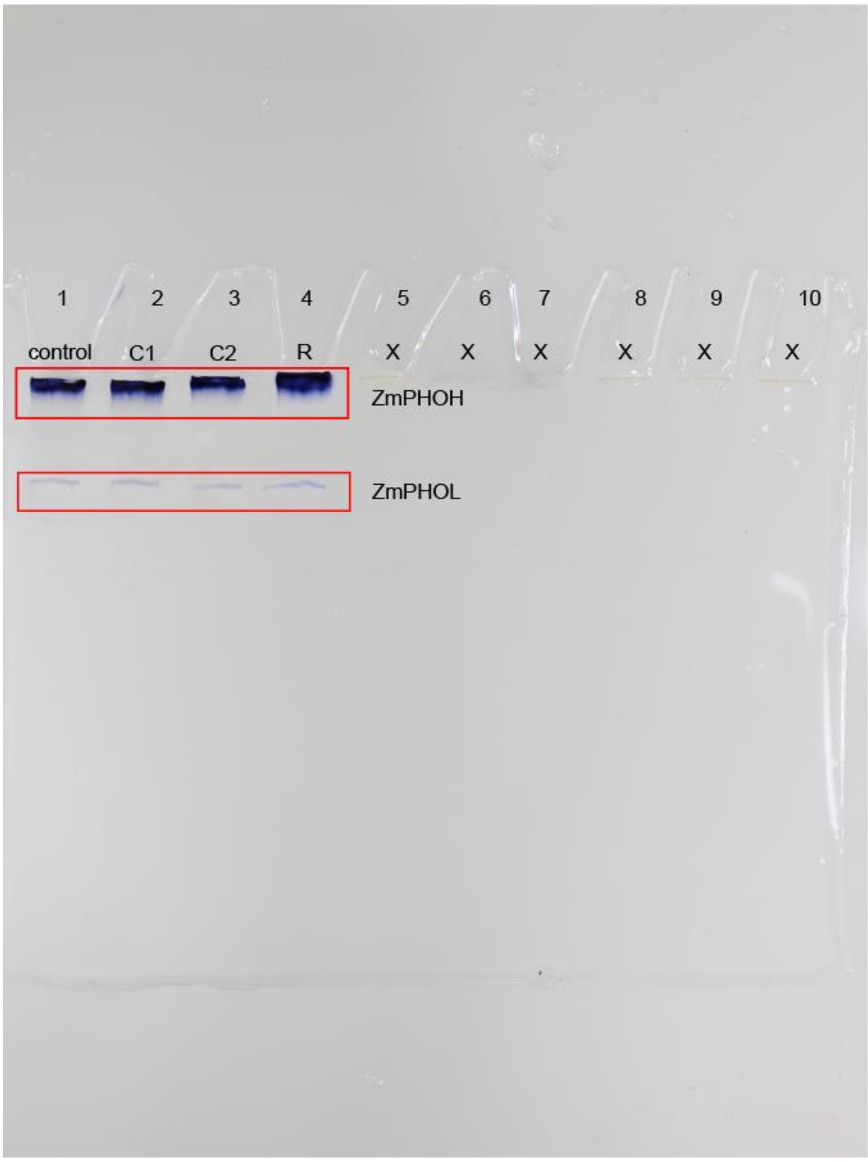

Figure S3 The whole gel image for the Native PAGE

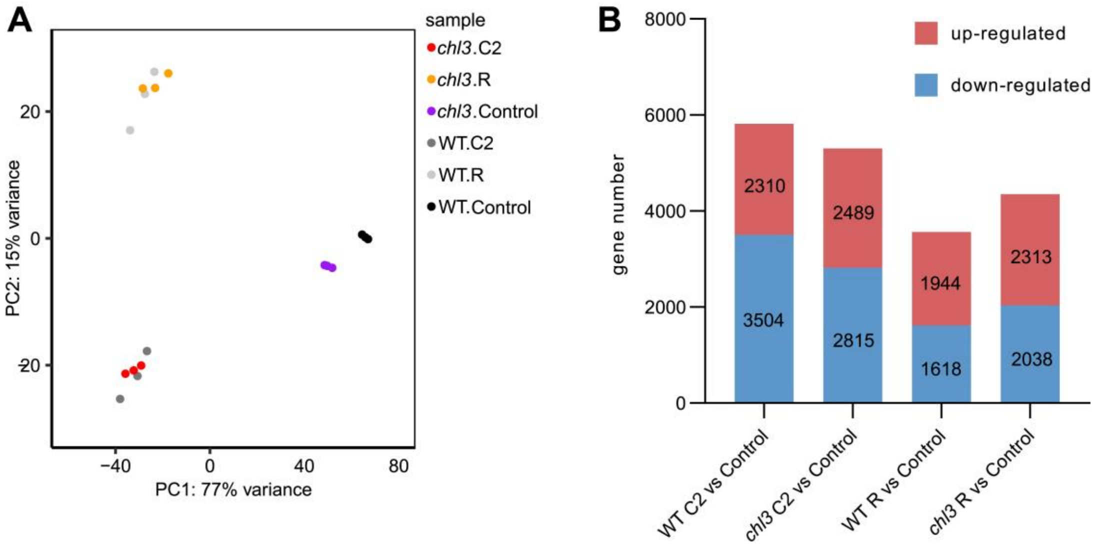

Figure S4 Statistical analysis of transcriptome data from wild-type and *chl3* young leaves during cold treatment and post-cold recovery

(A) Principal component analysis. (B) Statistical analysis of the number of differential genes in *chl3* and wild-type young leaves during cold treatment and post-cold recovery relative to pre-treatment. Control, before the start of cold treatment; C2, 48 h after cold treatment; R, 48 h after temperature recovery. WT, wild-type.

Table S1. P-value of changes in carbohydrates and carbohydrate derivatives.

		C1 vs Control	C2 vs Control	R vs Control
Maltose	WT	0.0012	0.025	0.28
	chl3	0.026	0.019	0.037
Fructose	WT	0.031	0.046	0.23
	chl3	0.011	0.0089	0.027
Glucose	WT	0.039	0.22	0.034
	chl3	0.0081	0.0065	0.0014
UDP-glucose	WT	0.51	0.06	0.68
	chl3	0	0.0084	0.041
Sucrose	WT	0.031	0.034	0.78
	chl3	0.012	0.0093	0.69
Galactose	WT	0.036	0.02	0.36
	chl3	0.0013	0.0015	0.026
Galactinol	WT	0.0024	0.24	0.048
	chl3	0.003	0.041	0.88
Raffinose	WT	0.00031	0.00098	0.002
	chl3	0.022	0.00093	0.00029
Stachyose	WT	0.018	0.0011	0.046
	chl3	0.029	0.036	0.027
Trehalose	WT	0.028	0.022	0.61
	chl3	0.0083	0.039	0.42

Trehalose 6-phosphate	WT	0.021	0.023	0.56
	chl3	0.00044	0.039	0.0007
Inositol	WT	0.0025	0.34	0.0041
	chl3	0.00043	0.029	0.0025

Table S2. The primers used in the study.

Primer	Sequence	Purpose
Actin-F	CCAAGGCCAACAGAGAGAAA	qRT-PCR
Actin-R	CCAAACGGAGAATAGCATGAG	
842-qF	GATGGTCAATATGAGTCAGC	qRT-PCR
842-qR	GAAGTGATGCGCTGCAAAGG	
